# Exploring the pathogenesis linking traumatic brain injury and epilepsy *via* bioinformatic analyses

**DOI:** 10.3389/fnagi.2022.1047908

**Published:** 2022-11-10

**Authors:** Gengshui Zhao, Yongqi Fu, Chao Yang, Xuehui Yang, Xiaoxiao Hu

**Affiliations:** ^1^Department of Neurosurgery, The People’s Hospital of Hengshui City, Hengshui, China; ^2^Department of Endocrinology, The People’s Hospital of Hengshui City, Hengshui, China; ^3^Department of Orthopedics, The People’s Hospital of Hengshui City, Hengshui, China

**Keywords:** bioinformatics, epilepsy, post-traumatic epilepsy, traumatic brain injury, interferon gamma

## Abstract

Traumatic brain injury (TBI) is a serious disease that could increase the risk of epilepsy. The purpose of this article is to explore the common molecular mechanism in TBI and epilepsy with the aim of providing a theoretical basis for the prevention and treatment of post-traumatic epilepsy (PTE). Two datasets of TBI and epilepsy in the Gene Expression Omnibus (GEO) database were downloaded. Functional enrichment analysis, protein–protein interaction (PPI) network construction, and hub gene identification were performed based on the cross-talk genes of aforementioned two diseases. Another dataset was used to validate these hub genes. Moreover, the abundance of infiltrating immune cells was evaluated through Immune Cell Abundance Identifier (ImmuCellAI). The common microRNAs (miRNAs) between TBI and epilepsy were acquired *via* the Human microRNA Disease Database (HMDD). The overlapped genes in cross-talk genes and target genes predicted through the TargetScan were obtained to construct the common miRNAs–mRNAs network. A total of 106 cross-talk genes were screened out, including 37 upregulated and 69 downregulated genes. Through the enrichment analyses, we showed that the terms about cytokine and immunity were enriched many times, particularly interferon gamma signaling pathway. Four critical hub genes were screened out for co-expression analysis. The miRNA–mRNA network revealed that three miRNAs may affect the shared interferon-induced genes, which might have essential roles in PTE. Our study showed the potential role of interferon gamma signaling pathway in pathogenesis of PTE, which may provide a promising target for future therapeutic interventions.

## Introduction

Traumatic brain injury (TBI) is a serious and challenging public health problem. In Europe more than 80,000 people die from TBI each year (Huijben et al., 2020), and the death toll is 53,000 in the United States (Samuels et al., 2019). TBI greatly increases the risk of epilepsy, and is a major cause of acquired epilepsy (Lutkenhoff et al., 2020). The prevalence of post-traumatic epilepsy (PTE) is unclear, with incidence ranging widely from 1.3 to 53.3% in prior studies (Xu et al., 2017; Wang et al., 2020). PTE not only affects patients’ quality of life, but also places a substantial economic burden for both families and society in general.

As the survival rate of TBI patients continues to increase, more patients are exposed to the risk of PTE. In the initial period of treatment, accurate identification of patients with high risk of epilepsy could be useful in epilepsy control, and may improve the quality of life for survivors (Wang et al., 2021).

So far, the precise molecular mechanisms that link TBI with epilepsy remain elusive. Current researches of TBI and epilepsy are concentrated mainly in retrospective observational studies and rodent models (Wang et al., 2020; Di Sapia et al., 2021). Previous studies have revealed that a sequence of molecular and cellular events may result in epilepsy after TBI, and pointed out the need for more valuable predictive and therapeutic biomarkers (Golub and Reddy, 2022). For high-risk patients, preventive or therapeutic measures in the latency between TBI and epilepsy onset may have an important impact on long-term outcomes (Di Sapia et al., 2021). Limited progress in understanding of neural networks and molecular pathologies has hampered personalized treatment, and new drug development (Dean et al., 2020). New methods are needed to obtain the comprehensive understanding of the disease pathophysiology at the molecular level to improve the precision and effectiveness of clinical interventions (Srinivasan and Brafman, 2021).

The rapid development of sequencing technologies informs new approaches for exploration of pathophysiological processes in TBI and epilepsy. This study explored the related genes and pathways in the pathogenesis of the two neurological diseases. To our knowledge, this is the first study to investigate the shared genes and key pathways in TBI and epilepsy *via* bioinformatic methodology. These findings may inspire new ideas for prediction and treatment of PTE.

## Materials and methods

### Datasets and study design

**FIGURE 1:**
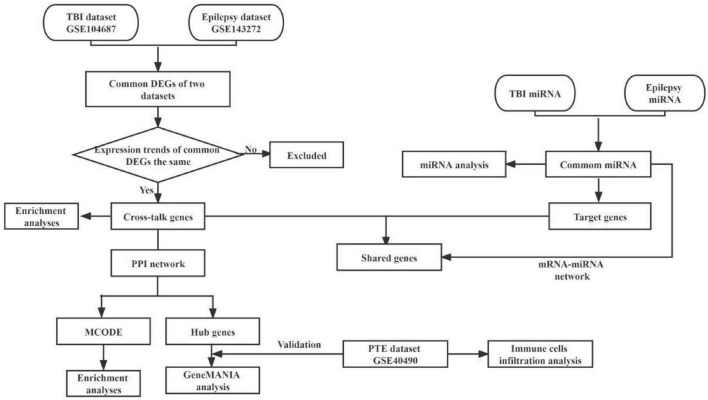
The workflow of present bioinformatics analysis. TBI, traumatic brain injury; DEGs, differentially expressed genes; GO, gene ontology; KEGG, Kyoto Encyclopedia of Genes and Genomes; PPI, protein–protein interactions; MCODE, molecular complex detection; PTE, post-traumatic epilepsy.

We used the key word “traumatic brain injury” and “epilepsy” to search TBI and epilepsy gene expression data from the Gene Expression Omnibus (GEO)^1^ and published researches. Two microarray datasets GSE104687 (Miller et al., 2017; Ma et al., 2021) and GSE143272 (Rawat et al., 2020) were downloaded, and were regarded as the discovery cohort. The GSE104687 dataset contains 93 TBI samples and 103 samples without TBI, and GSE143272 consists of 34 samples with epilepsy and 50 healthy samples as the control group. A diagram of the analytical workflow was described in Figure 1.

### Identification of potential cross-talk genes

NetworkAnalyst^2^ is an online R-based analysis tool (Zhou et al., 2019). We used NetworkAnalyst to compare gene expression profiles between different groups in GSE143272. Genes with a *p*-value < 0.05 were deemed as epileptic differentially expressed genes (DEGs). Meanwhile, the DEGs about TBI were obtained. The Venn diagram was used to identify the common DEGs from both datasets by using an online analysis platform.^3^ Subsequently, the DEGs with the same expression trend in GSE104687 and GSE143272 were regarded as cross-talk genes. These genes linking TBI and epilepsy were analyzed further.

### Functional enrichment analysis

To explore the biological significance of the cross-talk genes, we performed Gene Ontology (GO) and Kyoto Encyclopedia of Genes and Genomes (KEGG) pathway enrichment analysis. KOBAS^4^ is a widely-used online server for gene functional enrichment (Bu et al., 2021). The enrichment analyses of KEGG and GO were performed in the KOBAS-i database. In this study, unless otherwise noted, the p-value < 0.05 was considered significant.

### Protein–protein interaction network construction, module analysis and selection of hub genes

Protein–protein interaction (PPI) network was investigated online by Search Tool for the Retrieval of Interacting Genes (STRING^5^). We used all STRING interaction sources with a minimum interaction score of 0.15. Then, Cytoscape (version 3.6.1)^6^ was used to visualize the network (Otasek et al., 2019). The plug-in Molecular Complex Detection (MCODE) was used to screen key gene modules in the PPI network. MCODE was run with default settings. Subsequently, the GO and KEGG enrichment analyses of modular genes were performed with KOBAS-i. We used the Cytoscape plug-in CytoHubba to screen hub genes from cross-talk genes. The overlapped genes were considered as hub genes according to the score calculated by three common algorithms, namely Maximal Clique Centrality (MCC), Maximum Neighborhood Component (MNC), and EcCentricity.

### Validated cohort: Analyses of hub genes expression

To further clarify the expression of these hub genes in PTE, we found another public dataset (GSE40490). The dataset provided gene expression profiles in the traumatic epilepsy model of male Wistar rats. Four induced epilepsy samples (confirmed by epileptiform discharge monitoring on day five) and four control samples were included in this dataset. Comparisons between two groups were performed with the *t*-test. Moreover, a co-expression network of critical hub genes was constructed through GeneMANIA,^7^ which is an open source web platform to analysis gene lists.

### Immune infiltration analysis

The Immune Cell Abundance Identifier (ImmuCellAI) is an analytical tool to estimate the abundance of 24 immune cells including 18 T-cell subtypes and 6 other immune cells (Miao et al., 2020). We used ImmuCellAI to quantify the infiltration levels of immune cell types in the aforementioned two different groups in GSE40490.

### Identified the common microRNAs in traumatic brain injury and epilepsy

MicroRNAs (miRNAs), non-coding RNA molecules, have been reported to play key roles in regulation of target gene expression (Leitão and Enguita, 2022). To clarify whether some miRNAs could regulate cross-talk genes in TBI and epilepsy pathologies, we further researched the potential miRNAs. The Human microRNA Disease Database (HMDD)^8^, a reliable database, could provide experiment-based evidences for human miRNA and disease associations. TBI-related and epilepsy-related miRNAs were screened *via* HMDD. The overlapped miRNAs were obtained. In order to investigate the interactions and functions of miRNAs, we used the miRNA Enrichment Analysis and Annotation Tool (miEAA^9^) to conduct KEGG pathway analysis.

### The miRNAs–mRNAs network construction

We uesd TargetScan^10^ to predicte target genes of aforementioned miRNAs. The overlapped of target genes of common miRNAs and cross-talk genes in TBI and epilepsy were obtained, and they were used to construct the miRNAs–mRNAs regulated network. The output was visualized using Cytoscape.

## Results

### Identification of cross-talk genes

**FIGURE 2:**
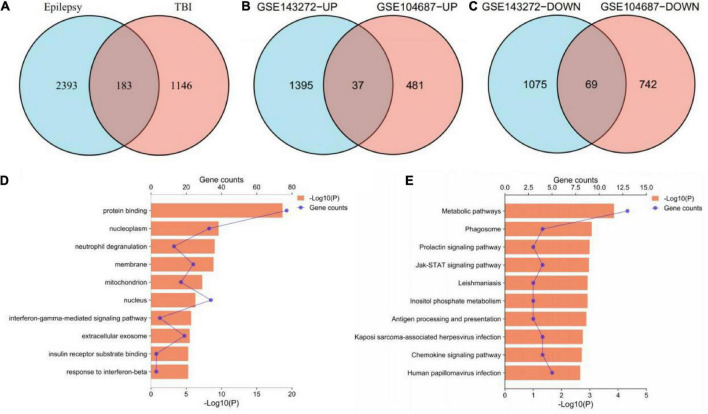
Identification and functional enrichment of cross-talk genes. **(A)** 183 common DEGs. **(B)** 37 upregulated genes. **(C)** 69 downregulated genes. **(D)** GO terms of cross-talk genes (top 10 terms were listed). **(E)** KEGG terms of cross-talk genes (top 10 terms were listed). DEGs, differentially expressed genes; GO, gene ontology; KEGG, Kyoto Encyclopedia of Genes and Genomes.

According to the aforementioned screening criteria, 1,329 DEGs were obtained in GSE104687 (TBI), and 2,576 DEGs were obtained in GSE143272 (epilepsy). These DEGs were subjected to Venn diagram analysis, and 183 common DEGs were identified (Figure 2A). The common DEGs with opposite expression trends were excluded, and we obtained 106 cross-talk genes including 37 upregulated and 69 downregulated genes in GSE104687 and GSE143272 (Figures 2B–C and Supplementary Table 1).

### Functional enrichment of cross-talk genes

In terms of GO analysis, cross-talk genes were mainly enriched in protein binding (*P* = 2.18878E-19), nucleoplasm (*P* = 2.62157E-10) and interferon-gamma-mediated signaling pathway (*P* = 2.04642E-06) (Figure 2D). The significant enriched KEGG terms contained metabolic pathways (*P* = 0.000140913), Jak-STAT signaling pathway (*P* = 0.001085932) and chemokine signaling pathway (*P* = 0.001922808) (Figure 2E). These results indicated that chemokines and cytokines may play important roles in the pathological processes of these two neurological diseases.

### Protein–protein interaction network construction, module analysis and selection of hub genes

**FIGURE 3:**
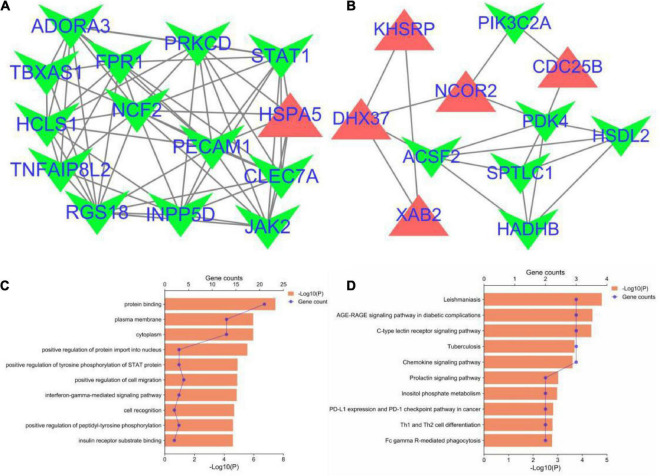
Significant gene module and enrichment analysis of the modular genes. **(A,B)** Top two significant gene clustering modules. Red indicates upregulated genes and green indicates downregulated genes. **(C,D)** GO and KEGG enrichment analysis of the modular genes (top 10 terms of each category were listed).

**FIGURE 4:**
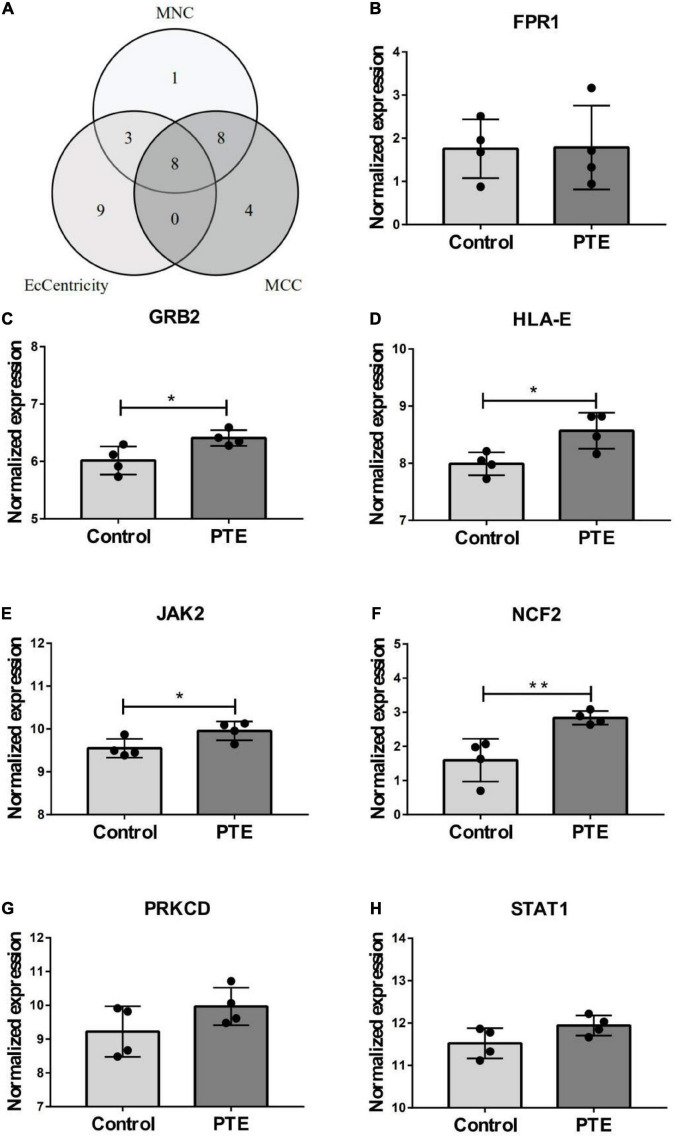
Identification and expression level of hub gene. **(A)** The Venn diagram of three algorithms. **(B–H)** The expression level of hub gene in GSE40490. The comparison between the two sets of data uses the mean *t*-test. *p < 0.05; **p < 0.01.

The PPI network of the cross-talk genes was constructed using STRING and Cytoscape, which contains 105 nodes and 390 edges (Supplementary Figure 1). Top two highly interconnected clusters of gene modules were identified by the MCODE with default cutoffs. A total of 25 nodes and 80 edges were included in these modules (Figures 3A,B). GO analysis showed that these genes were still involved in interferon-gamma-mediated signaling pathway (*P* = 1.43398E-05) (Figure 3C). Similarly, chemokine signaling pathway remains in the top 10 most significant terms in the KEGG pathway analysis (*P* = 0.000248226) (Figure 3D). Top 20 hub genes identified by aforementioned three methods in cytoHubba. Then, the venn diagram showed the intersecting genes in different algorithms, including CLEC7A, JAK2, PRKCD, STAT1, FPR1, HLA-E, NCF2 and GRB2 (Figure 4A). The detailed description of the top 20 genes ranked in cytoHubba was provided in Table 1.

**TABLE 1:** The top 20 genes ranked in cytoHubba.

MNC	EcCentricity	MCC
HSPA5	**CLEC7A**	**NCF2**
**JAK2**	NR2C2	PECAM1
**STAT1**	HSDL2	**CLEC7A**
PECAM1	PDK4	**JAK2**
DNMT1	TRIB2	**STAT1**
**PRKCD**	TSC22D3	RGS18
**NCF2**	**FPR1**	**FPR1**
INPP5D	STOM	INPP5D
RGS18	SVIL	HCLS1
**GRB2**	HADHB	ADORA3
**CLEC7A**	PPRC1	CSF2RA
NR2C2	**GRB2**	**PRKCD**
NCOR2	**JAK2**	RNASE2
HCLS1	NCOR2	**GRB2**
PDK4	**STAT1**	HSPA5
**FPR1**	**PRKCD**	IGSF6
CSF2RA	HLA-B	TNFAIP8L2
ETS1	**ACSF2**	TBXAS1
ADORA3	**HLA-E**	**HLA-E**
**HLA-E**	NCF2	DNMT1

Bold gene symbols were the overlap hub genes in top 20 by three ranked methods, respectively in cytoHubba. MNC: maximum neighborhood component; MCC: maximal clique centrality.

### Validated cohort: The differential genes analysis in post-traumatic epilepsy

In order to investigate the stability of these hub gene expression levels, we analyzed the expression of these genes in GSE40490. We transformed related rats genes into homologous human genes based on NCBI’s HomoloGene database.^11^ The expressions of the seven genes (the expression value of CLEA7A missing in GSE40490) in different groups were shown in Figures 4B–H. Combining with the histogram results, four critical hub genes further were screened out. These results indicated that GRB2, JAK2, HLA-E and NCF2 may be closely related to the occurrence and development of PTE. Detailed information about critical hub genes were listed in Table 2.

**TABLE 2:** The details of the validated hub genes.

No.	Gene symbol	Description	Function
1	GRB2	Growth factor receptor bound protein 2	The protein encoded by this gene binds the epidermal growth factor receptor and contains one SH2 domain and two SH3 domains. Among its related pathways contain Cytokine Signaling in Immune system
2	JAK2	Janus kinase 2	This gene encodes a non-receptor tyrosine kinase that plays a central role in cytokine and growth factor signaling. This gene is a downstream target of the pleiotropic cytokine IL6 that is produced by B cells, T cells, dendritic cells and macrophages to produce an immune response or inflammation
3	HLA-E	Major histocompatibility complex, class I, E	HLA-E belongs to the HLA class I heavy chain paralogues. HLA-E binds a restricted subset of peptides derived from the leader peptides of other class I molecules
4	NCF2	Neutrophil cytosolic factor 2	NCF2 is a Protein Coding gene. Among its related pathways are Class I MHC mediated antigen processing and presentation and G-protein signaling RAC1 in cellular process

**FIGURE 5:**
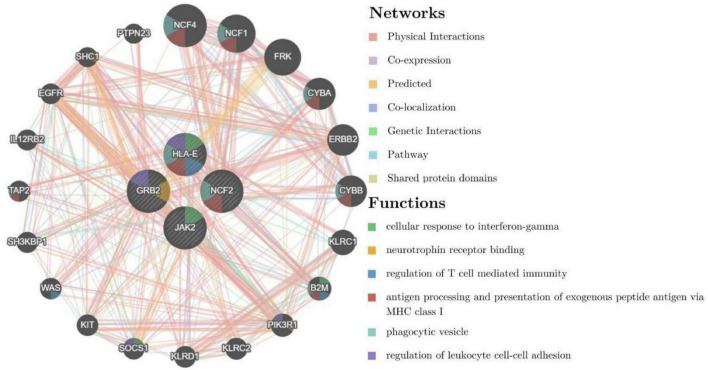
Co-expression network of critical hub genes.

To further explore the critical hub genes, we performed the functional interaction networks using the GeneMANIA database. These genes showed a complex PPI network with physical interactions of 77.64%, co-expression of 8.01%, prediction of 5.37%, co-localization of 3.63%, genetic interactions of 2.87%, pathway of 1.88% and shared protein domains 0.60%. Functional analysis revealed that these genes were involved in cellular response to interferon-gamma, neurotrophin receptor binding and regulation of T cell mediated immunity (Figure 5). The comparison of enrichment terms in the discovery cohort and the validation cohort increased the reliability of our findings in the discovery cohort.

### Immune cell infiltration

**FIGURE 6:**
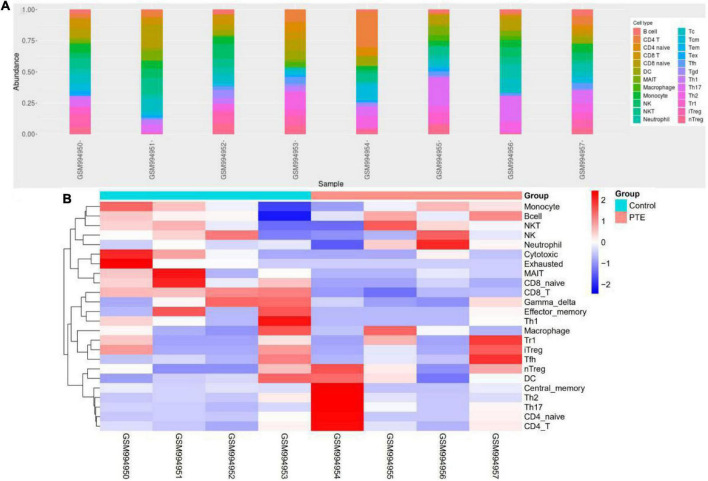
Immune profile of GSE40490 through ImmuCellAI. **(A)** Abundance of 24 immune cell types in GSE40490. **(B)** Differences between the cell types and individual samples.

ImmuCellAI was performed to further determine the abundance of 24 immune cell types in different groups. A heatmap with hierarchical clustering was generated to reveal the differences between the cell types and individual samples (Figure 6). We found that the abundance of CD8 T cells and CD8 naive T cells (both *p* = 0.03) were significantly lower in the PTE group (Table 3 and Supplementary Figure 2).

**TABLE 3:** Comparisons between immune cells in two groups.

Immune cell	Composition (PTE)	Composition (Control)	*P*-value
DC	0.1135	0.0705	0.89
Bcell	0.097	0.0885	0.69
Monocyte	0.1435	0.1545	1
Macrophage	0.0425	0.041	0.89
NK	0.0565	0.1095	0.89
Neutrophil	0.15	0.1035	0.34
CD4_T	0.1305	0.0685	0.34
**CD8_T**	**0.042**	**0.173**	**0.03**
NKT	0.0895	0.0885	1
Gamma_delta	0.0255	0.112	0.2
CD4_naive	0.0055	5.00E-04	0.64
Tr1	0.0035	0.0025	0.64
nTreg	0.0075	0.0025	0.3
iTreg	0.001	0.003	1
Th1	0	0.0025	0.16
Th2	0.0065	0.0015	0.31
Th17	0.018	0.006	0.2
Tfh	0.002	0.0015	0.88
**CD8_naive**	**5.00E-04**	**0.0115**	**0.03**
Cytotoxic	5.00E-04	0.005	0.3
Exhausted	0	0.001	0.07
MAIT	5.00E-04	0.004	0.18
Central_memory	0.0065	0.008	1
Effector_memory	0	0.0015	0.4

Bold immune cells are significantly lower in the PTE group.

### Identified and analysis of common miRNAs in traumatic brain injury and epilepsy

**FIGURE 7:**
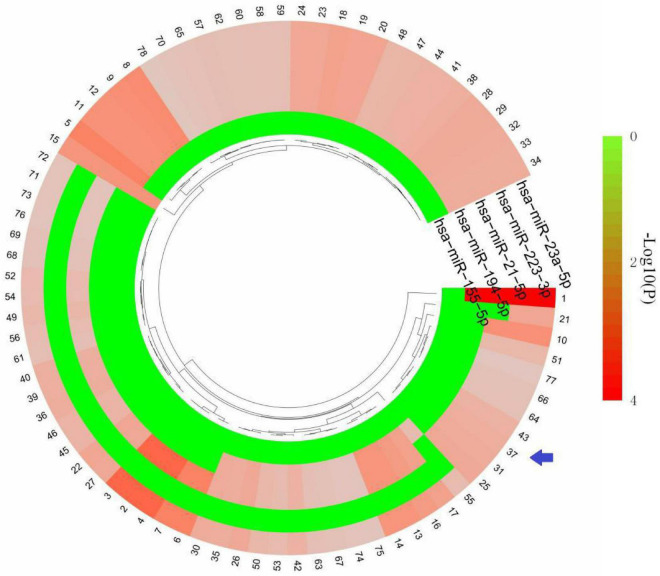
KEGG terms of five common miRNAs. The arrow indicates interferon gamma signaling pathway. The detailed description of each term is in Supplementary Table 3.

Based on the HMDD database,32 miRNAs were obtained to be associated with TBI and 25 miRNAs were associated with epilepsy (Supplementary Table 2). There were five common miRNAs (hsa-miR-155-5p, hsa-miR-194-5p, hsa-miR-21-5p, hsa-miR-223-3p, hsa-miR-23a-5p) in TBI and epilepsy. Subsequently, we obtained 78 significantly enriched pathways *via* KEGG analysis. Notably, many of the enriched terms were associated with immune response, and the interferon gamma signaling pathway was still enriched with a relative low *P* value (*P* = 0.0213079). It indicated the common miRNAs involved in the pathogenesis of TBI and epilepsy were also closely related to the interferon gamma signaling pathway (Figure 7 and Supplementary Table 3). That was consistent with our previous enrichment analysis once again.

### miRNAs–mRNAs regulation network

**FIGURE 8:**
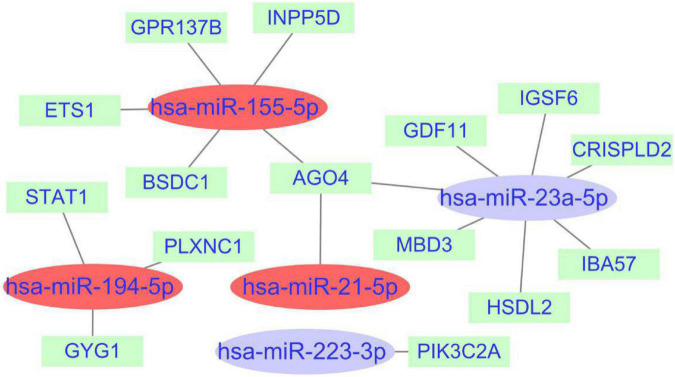
miRNAs-shared genes regulatory network. The ovals represent miRNAs and squares represent shared genes. The red ovals represent miRNAs associated with interferon gamma signaling pathway.

A total of 1,968 target genes of common miRNAs were predicted *via* TargetScan. After intersecting these genes with the cross-talk genes in discovery cohort, 15 shared genes were obtained. Finally, the miRNAs–mRNAs network was constructed, including 20 nodes (five miRNAs, 15 mRNAs) and 18 edges (Figure 8). The genes about interferon gamma signaling pathway were regulated by three miRNAs, namely hsa-miR-155-5p, hsa-miR-194-5p, and hsa-miR-21-5p. We hypothesized that the effects of miRNAs on PTE may be partly attributable to interferon gamma.

## Discussion

In this study, we explored the shared genes and key pathways in TBI and epilepsy, which might provide new insights into the pathophysiological mechanism of PTE. Moreover, to some extent, our findings may offer novel targets for identification of high-risk individuals, early prevention of secondary complications and drug discovery.

Previous studies have shown that JAK2 is involved in the pathological process of PTE (Klein et al., 2018; Hixson et al., 2019), and hsa-miR-155-5p plays an important role in epileptic progression (Li-Gang Huang, 2018; Yu et al., 2021). Those data are consistent with our findings. However, those researches have targeted a specific single gene or pathway, which limits deeper understanding of the underlying mechanisms. This study performed a comprehensive exploration of cross-talk mechanisms between the TBI and epilepsy. The results of four functional enrichment analyses were basically consistent, including three different gene sets and one miRNA set. That may suggest the stability of our findings. Through the enrichment analyses, we showed that the terms about cytokine and immunity were enriched many times, particularly interferon-gamma-mediated signaling pathway. Interferon gamma is likely to play a pivotal role in the pathological process of PTE.

Interferon gamma is mainly involved in immune regulation, and its expression level is associated with neurological diseases (Zhang et al., 2020; Döhne et al., 2022; Wen et al., 2022). TBI could induce a serial of cytokines, including interferon gamma and activate various types of immune cells (Siwicka-Gieroba and Dabrowski, 2021; Salem et al., 2022). A published study on status epileptics suggests that intraventricular injection of recombinant interferon gamma may reduce neuron damage on rat hippocampus (Ryu et al., 2010). A clinical study including 254 patients with TBI analyzed interferon gamma levels in blood. The levels of interferon gamma in PTE gruop were higher than non-seizure group (Choudhary et al., 2021). Thus, we conjecture that interferon gamma may act as a bridge from TBI to epilepsy.

Although most of the researches on immune cell infiltration focuses on the field of tumors, some scholars have explored the influences of immune system in neurological diseases, including PTE. Several recent studies have shown that the strong immune response after TBI triggers a cascade of inflammatory cells and cytokines, which may be responsible for epilepsy (Sharma et al., 2019). Activation of immune signaling pathways may result in the loss of GABaergic neurons in the hippocampus, leading to reduction in synaptic inhibition and a lower seizure threshold (Webster et al., 2017). There is also evidence that the number of CD8 T cells in the hippocampus is related to the degree of neuronal loss (Lu et al., 2017; Langenbruch et al., 2020). Moreover, CD8 T cells could produce interferon gamma (De Benedetti et al., 2021). We hypothesize CD8 T cell may play a key role in the progression of PTE *via* interferon gamma, the important regulator of immune responses.

In our study, we analyzed the histogram results generated by the PTE dataset and further screened out four critical hub genes. These genes are all involved in the immune response. Several studies on other diseases have revealed the associations of hub genes expression with interferon gamma release (Johnson et al., 2020; Meng et al., 2021). Yet, in-depth mechanism studies on PTE about these hub genes and interferon gamma are needed. Among the seven histogram results, four critical hub genes further were screened out. We believe that the expression values of some genes do not meet the criteria of statistical significance may be related to the following factors: the limited sample size (four PTE samples) and short detection time (5 days after induction).

MicroRNAs (miRNAs), a group of small non-coding RNAs, are involved in pathological mechanisms of a variety of neurological diseases, and influence their prognoses (Xie et al., 2022; Ünalp et al., 2022). Five miRNAs were screened out in this study, and miRNA-gene network constructed may help to understand the common mechanisms of PTE. A recent study indicated that miRNAs have the potential to enhance functional recovery after TBI by promoting neurogenesis and axonal growth (Yang et al., 2022). MiRNAs could affect protein expression by changing mRNA folding or reducing mRNA stability. This pathological alteration may play an important role in the pathogenesis of drug-resistant epilepsy (Srivastava et al., 2016).

Despite the promising results we showed, there are also limitations in our study. Using public database, we revealed the important role for interferon gamma signaling pathway in the pathological processes associated with TBI and epilepsy, yet explanations are mostly speculative. Our findings need to be further validated in disease models *in vitro* and *in vivo*, especially applying Tbx21 knockout mice. Those mice lack TBX21, a critical transcription factor for the control of interferon gamma production. This will be the focus of our future efforts (Raposo et al., 2014).

## Conclusion

Our study showed the potential role of interferon gamma signaling pathway in pathogenesis of PTE, which may provide a promising target for future therapeutic interventions.

## Data availability statement

The original contributions presented in this study are included in the article/Supplementary material, further inquiries can be directed to the corresponding author.

## Author contributions

GZ and XY had the idea for the article. GZ, YF, XH, and CY analyzed the data. GZ and YF performed the data correction and image rendering. GZ wrote the manuscript. XY critically revised the work. All authors have read and approved the final manuscript.

## Footnotes

1
http://www.ncbi.nlm.nih.gov/geo/

2
https://www.networkanalyst.ca/

3
https://www.bioinformatics.com.cn

4
http://kobas.cbi.pku.edu.cn

5
http://string-db.org

6
https://cytoscape.org/

7
http://genemania.org

8
http://www.cuilab.cn/hmdd

9
https://ccb-compute2.cs.uni-saarland.de/mieaa2/

10
https://www.targetscan.org

11
https://www.ncbi.nlm.nih.gov/homologene

## Supplementary material

Supplementary Figure 1 Protein–protein interactions network of the cross-talk genes red indicates upregulated genes, and green indicates downregulated genes.

Supplementary Figure 2 Immune cell comparisons between PTE group and control group. *P*-value < 0.05 is considered statistically significant. The blue box plot indicates PTE group and the red box plot represents contRMAT 4.

## Abbreviations

TBI: traumatic brain injury
PTE: Post-traumatic epilepsy
GEO: Gene Expression Omnibus
DEGs: differentially expressed genes
GO: Gene Ontology
KEGG: Kyoto Encyclopedia of Genes and Genomes
PPI: protein–protein interactions
STRING: search tool for the retrieval of interacting genes
MCODE: molecular complex detection
MCC: maximal clique centrality
MNC: maximum neighborhood component
ImmuCellAI: immune cell abundance identifier
miRNA: microRNA
HMDD: human microRNA disease database
miEAA: miRNA enrichment analysis and annotation tool.
