# Supplementary material for: Exploring the pathogenesis linking traumatic brain injury and epilepsy via bioinformatic analyses
Source: Front Aging Neurosci. 2022 Nov 10;14:1047908. doi: 10.3389/fnagi.2022.1047908 (PMC9686289; doi:10.3389/fnagi.2022.1047908)
Supplement: Supplementary file 1 [file Table_1.DOCX]

**Supplemental Table 1.** 106 cross-talk genes

| **Gene** | **Description** | **Up/Down** |
| --- | --- | --- |
| XAB2 | XPA binding protein 2 | Up |
| PELP1 | "proline, glutamate and leucine rich protein 1" | Up |
| CDC25B | cell division cycle 25B | Up |
| USE1 | unconventional SNARE in the ER 1 | Up |
| NCOR2 | nuclear receptor corepressor 2 | Up |
| CCDC137 | coiled-coil domain containing 137 | Up |
| CECR7 | "cat eye syndrome chromosome region, candidate 7" | Up |
| DDX39A | DExD-box helicase 39A | Up |
| LRP5L | LDL receptor related protein 5 like | Up |
| SLC38A2 | solute carrier family 38 member 2 | Up |
| IBA57 | iron-sulfur cluster assembly factor IBA57 | Up |
| DHX37 | DEAH-box helicase 37 | Up |
| CXXC5 | CXXC finger protein 5 | Up |
| EPC1 | enhancer of polycomb homolog 1 | Up |
| VARS2 | "valyl-tRNA synthetase 2, mitochondrial" | Up |
| DBP | D-box binding PAR bZIP transcription factor | Up |
| TRMT1 | tRNA methyltransferase 1 | Up |
| ZDHHC8 | zinc finger DHHC-type palmitoyltransferase 8 | Up |
| LIME1 | Lck interacting transmembrane adaptor 1 | Up |
| DNMT1 | DNA methyltransferase 1 | Up |
| HSPA5 | heat shock protein family A (Hsp70) member 5 | Up |
| CEP85L | centrosomal protein 85 like | Up |
| MTFP1 | mitochondrial fission process 1 | Up |
| ETS1 | "ETS proto-oncogene 1, transcription factor" | Up |
| GDF11 | growth differentiation factor 11 | Up |
| KHSRP | KH-type splicing regulatory protein | Up |
| MZF1 | myeloid zinc finger 1 | Up |
| PPRC1 | PPARG related coactivator 1 | Up |
| POLR3H | RNA polymerase III subunit H | Up |
| GTPBP6 | GTP binding protein 6 (putative) | Up |
| MBD3 | methyl-CpG binding domain protein 3 | Up |
| SCRIB | scribble planar cell polarity protein | Up |
| ZNF30 | zinc finger protein 30 | Up |
| ENGASE | endo-beta-N-acetylglucosaminidase | Up |
| NDUFS7 | NADH:ubiquinone oxidoreductase core subunit S7 | Up |
| TRIB2 | tribbles pseudokinase 2 | Up |
| REPIN1 | replication initiator 1 | Up |
| MTM1 | myotubularin 1 | Down |
| SPTLC1 | serine palmitoyltransferase long chain base subunit 1 | Down |
| COX15 | cytochrome c oxidase assembly homolog COX15 | Down |
| FHL3 | four and a half LIM domains 3 | Down |
| GPR137B | G protein-coupled receptor 137B | Down |
| TMIGD3 | transmembrane and immunoglobulin domain containing 3 | Down |
| HLA-E | "major histocompatibility complex, class I, E" | Down |
| GRB2 | growth factor receptor bound protein 2 | Down |
| DYNLT1 | dynein light chain Tctex-type 1 | Down |
| NCF2 | neutrophil cytosolic factor 2 | Down |
| RNASE2 | ribonuclease A family member 2 | Down |
| TSC22D3 | TSC22 domain family member 3 | Down |
| PRKCD | protein kinase C delta | Down |
| UBE2L6 | ubiquitin conjugating enzyme E2 L6 | Down |
| PLXNC1 | plexin C1 | Down |
| NR2C2 | nuclear receptor subfamily 2 group C member 2 | Down |
| ASAH1 | N-acylsphingosine amidohydrolase 1 | Down |
| PIK3C2A | phosphatidylinositol-4-phosphate 3-kinase catalytic subunit type 2 alpha | Down |
| ACSF2 | acyl-CoA synthetase family member 2 | Down |
| AGO4 | argonaute RISC component 4 | Down |
| SVIL | supervillin | Down |
| STOM | stomatin | Down |
| ATP8B4 | ATPase phospholipid transporting 8B4 (putative) | Down |
| JAK2 | Janus kinase 2 | Down |
| HAUS4 | HAUS augmin like complex subunit 4 | Down |
| TBXAS1 | thromboxane A synthase 1 | Down |
| ACOT9 | acyl-CoA thioesterase 9 | Down |
| BSDC1 | BSD domain containing 1 | Down |
| PNPLA8 | patatin like phospholipase domain containing 8 | Down |
| DNASE1L1 | deoxyribonuclease 1 like 1 | Down |
| MR1 | "major histocompatibility complex, class I-related" | Down |
| RGS18 | regulator of G protein signaling 18 | Down |
| PDK4 | pyruvate dehydrogenase kinase 4 | Down |
| STAT1 | signal transducer and activator of transcription 1 | Down |
| FPR1 | formyl peptide receptor 1 | Down |
| CD302 | CD302 molecule | Down |
| PRR13 | proline rich 13 | Down |
| PECAM1 | platelet and endothelial cell adhesion molecule 1 | Down |
| IFITM3 | interferon induced transmembrane protein 3 | Down |
| GDAP2 | ganglioside induced differentiation associated protein 2 | Down |
| ARL17B | ADP ribosylation factor like GTPase 17B | Down |
| DHRS12 | dehydrogenase/reductase 12 | Down |
| CSGALNACT1 | chondroitin sulfate N-acetylgalactosaminyltransferase 1 | Down |
| CLEC7A | C-type lectin domain containing 7A | Down |
| MGME1 | mitochondrial genome maintenance exonuclease 1 | Down |
| GPR34 | G protein-coupled receptor 34 | Down |
| PLSCR1 | phospholipid scramblase 1 | Down |
| OS9 | OS9 endoplasmic reticulum lectin | Down |
| EFEMP2 | EGF containing fibulin extracellular matrix protein 2 | Down |
| HADHB | hydroxyacyl-CoA dehydrogenase trifunctional multienzyme complex subunit beta | Down |
| INPP5D | inositol polyphosphate-5-phosphatase D | Down |
| OSBPL11 | oxysterol binding protein like 11 | Down |
| HLA-B | "major histocompatibility complex, class I, B" | Down |
| CPNE1 | copine 1 | Down |
| CSF2RA | colony stimulating factor 2 receptor subunit alpha | Down |
| PCTP | phosphatidylcholine transfer protein | Down |
| IGSF6 | immunoglobulin superfamily member 6 | Down |
| FEZ2 | fasciculation and elongation protein zeta 2 | Down |
| GYG1 | glycogenin 1 | Down |
| HSDL2 | hydroxysteroid dehydrogenase like 2 | Down |
| CYBRD1 | cytochrome b reductase 1 | Down |
| CRISPLD2 | cysteine rich secretory protein LCCL domain containing 2 | Down |
| CCDC126 | coiled-coil domain containing 126 | Down |
| TNFAIP8L2 | TNF alpha induced protein 8 like 2 | Down |
| RNASE4 | ribonuclease A family member 4 | Down |
| TP53BP2 | tumor protein p53 binding protein 2 | Down |
| ALPL | "alkaline phosphatase, biomineralization associated" | Down |
| HCLS1 | hematopoietic cell-specific Lyn substrate 1 | Down |
| ZNF224 | zinc finger protein 224 | Down |
