# Supplementary material for: Exploring the pathogenesis linking traumatic brain injury and epilepsy via bioinformatic analyses
Source: Front Aging Neurosci. 2022 Nov 10;14:1047908. doi: 10.3389/fnagi.2022.1047908 (PMC9686289; doi:10.3389/fnagi.2022.1047908)
Supplement: Supplementary file 2 [file Table_2.DOCX]

**Supplemental Table 2.**MiRNAs associated with epilepsy and TBI

| Class | MiRNA |
| --- | --- |
| Epilepsy | hsa-let-7i;hsa-mir-107;hsa-mir-126;hsa-mir-135b;hsa-mir-142;hsa-mir-150;hsa-mir-155;hsa-mir-16;hsa-mir-191;hsa-mir-194;hsa-mir-21;hsa-mir-223;hsa-mir-23a;hsa-mir-27b;hsa-mir-300;hsa-mir-30e;hsa-mir-3195;hsa-mir-328;hsa-mir-3610;hsa-mir-3945;hsa-mir-423;hsa-mir-450;hsa-mir-499;hsa-mir-598;hsa-mir-93 |
| TBI | hsa-let-7b;hsa-let-7d;hsa-mir-106b;hsa-mir-130a;hsa-mir-132;hsa-mir-134;hsa-mir-139;hsa-mir-146a;hsa-mir-155;hsa-mir-15a;hsa-mir-17;hsa-mir-181c;hsa-mir-184;hsa-mir-194;hsa-mir-21;hsa-mir-210;hsa-mir-221;hsa-mir-222;hsa-mir-223;hsa-mir-23a;hsa-mir-298;hsa-mir-301a;hsa-mir-30a;hsa-mir-30b;hsa-mir-320a;hsa-mir-323a;hsa-mir-342;hsa-mir-34a;hsa-mir-378;hsa-mir-4446;hsa-mir-451a;hsa-mir-9a |
