# Supplementary material for: Exploring the pathogenesis linking traumatic brain injury and epilepsy via bioinformatic analyses
Source: Front Aging Neurosci. 2022 Nov 10;14:1047908. doi: 10.3389/fnagi.2022.1047908 (PMC9686289; doi:10.3389/fnagi.2022.1047908)
Supplement: Supplementary file 3 [file Table_3.DOCX]

**Supplemental Table 3.**The KEGG analysis of five common miRNAs.

| No. | Pathway | P-value | miRNAs |
| --- | --- | --- | --- |
| 1 | Axon guidance mediated by Slit Robo | 4.00E-04 | hsa-miR-155-5p; hsa-miR-194-5p; hsa-miR-21-5p; hsa-miR-223-3p |
| 2 | Alanine biosynthesis | 0.0020664 | hsa-miR-155-5p; hsa-miR-21-5p |
| 3 | Leucine biosynthesis | 0.0020664 | hsa-miR-155-5p; hsa-miR-21-5p |
| 4 | Synthesis and Degradation of Ketone Bodies | 0.0020664 | hsa-miR-155-5p; hsa-miR-21-5p |
| 5 | Insulin IGF pathway mitogen activated protein kinase kinase MAP kinase cascade | 0.0047118 | hsa-miR-155-5p; hsa-miR-194-5p; hsa-miR-21-5p; hsa-miR-223-3p |
| 6 | Isoleucine biosynthesis | 0.0047947 | hsa-miR-155-5p; hsa-miR-21-5p |
| 7 | Valine biosynthesis | 0.0047947 | hsa-miR-155-5p; hsa-miR-21-5p |
| 8 | B cell activation | 0.0064481 | hsa-miR-155-5p; hsa-miR-194-5p; hsa-miR-21-5p; hsa-miR-223-3p |
| 9 | Alpha 6 Beta 4 signaling pathway | 0.0069458 | hsa-miR-155-5p; hsa-miR-194-5p; hsa-miR-21-5p; hsa-miR-223-3p |
| 10 | ACE Inhibitor Pathway | 0.0075382 | hsa-miR-155-5p; hsa-miR-21-5p |
| 11 | Long term depression | 0.0077435 | hsa-miR-155-5p; hsa-miR-194-5p; hsa-miR-21-5p; hsa-miR-223-3p |
| 12 | Type II diabetes mellitus | 0.0077435 | hsa-miR-155-5p; hsa-miR-194-5p; hsa-miR-21-5p; hsa-miR-223-3p |
| 13 | Osteopontin Signaling | 0.0088925 | hsa-miR-155-5p; hsa-miR-21-5p; hsa-miR-223-3p |
| 14 | SRF and miRs in Smooth Muscle Differentiation and Proliferation | 0.0088925 | hsa-miR-155-5p; hsa-miR-21-5p; hsa-miR-223-3p |
| 15 | EGF EGFR Signaling Pathway | 0.0090057 | hsa-miR-155-5p; hsa-miR-194-5p; hsa-miR-21-5p; hsa-miR-223-3p; hsa-miR-23a-5p |
| 16 | Selenium Metabolism and Selenoproteins | 0.0109678 | hsa-miR-155-5p; hsa-miR-21-5p; hsa-miR-223-3p |
| 17 | Cytosolic DNA sensing pathway | 0.0133203 | hsa-miR-155-5p; hsa-miR-21-5p; hsa-miR-223-3p |
| 18 | B cell receptor signaling pathway | 0.0135581 | hsa-miR-155-5p; hsa-miR-194-5p; hsa-miR-21-5p; hsa-miR-223-3p |
| 19 | Insulin IGF pathway protein kinase B signaling cascade | 0.0148379 | hsa-miR-155-5p; hsa-miR-194-5p; hsa-miR-21-5p; hsa-miR-223-3p |
| 20 | Epithelial cell signaling in Helicobacter pylori infection | 0.0148379 | hsa-miR-155-5p; hsa-miR-194-5p; hsa-miR-21-5p; hsa-miR-223-3p |
| 21 | Formyltetrahydroformate biosynthesis | 0.0161297 | hsa-miR-155-5p; hsa-miR-194-5p |
| 22 | Vasopressin synthesis | 0.0161297 | hsa-miR-155-5p; hsa-miR-21-5p |
| 23 | IL 4 signaling pathway | 0.0166751 | hsa-miR-155-5p; hsa-miR-194-5p; hsa-miR-21-5p; hsa-miR-223-3p |
| 24 | RANKL RANK Signaling Pathway | 0.0171585 | hsa-miR-155-5p; hsa-miR-194-5p; hsa-miR-21-5p; hsa-miR-223-3p |
| 25 | Axon guidance mediated by netrin | 0.0173933 | hsa-miR-155-5p; hsa-miR-194-5p; hsa-miR-21-5p |
| 26 | miRs in Muscle Cell Differentiation | 0.0173933 | hsa-miR-155-5p; hsa-miR-21-5p; hsa-miR-223-3p |
| 27 | Synthesis and degradation of ketone bodies | 0.0175957 | hsa-miR-155-5p; hsa-miR-21-5p |
| 28 | T cell activation | 0.018155 | hsa-miR-155-5p; hsa-miR-194-5p; hsa-miR-21-5p; hsa-miR-223-3p |
| 29 | Neural Crest Differentiation | 0.018155 | hsa-miR-155-5p; hsa-miR-194-5p; hsa-miR-21-5p; hsa-miR-223-3p |
| 30 | Base excision repair | 0.0189012 | hsa-miR-155-5p; hsa-miR-21-5p; hsa-miR-223-3p |
| 31 | Peptide GPCRs | 0.0191189 | hsa-miR-155-5p; hsa-miR-21-5p |
| 32 | Toll like receptor signaling pathway | 0.0191918 | hsa-miR-155-5p; hsa-miR-194-5p; hsa-miR-21-5p; hsa-miR-223-3p |
| 33 | Cytoskeletal regulation by Rho GTPase | 0.0197256 | hsa-miR-155-5p; hsa-miR-194-5p; hsa-miR-21-5p; hsa-miR-223-3p |
| 34 | Toll Like Receptor signaling | 0.0197256 | hsa-miR-155-5p; hsa-miR-194-5p; hsa-miR-21-5p; hsa-miR-223-3p |
| 35 | Notch signaling pathway | 0.0204862 | hsa-miR-155-5p; hsa-miR-21-5p; hsa-miR-223-3p |
| 36 | Glycosaminoglycan degradation | 0.0206986 | hsa-miR-155-5p; hsa-miR-21-5p |
| **37** | **Interferon gamma signaling pathway** | **0.0213079** | **hsa-miR-155-5p; hsa-miR-194-5p; hsa-miR-21-5p** |
| 38 | Ras Pathway | 0.02139 | hsa-miR-155-5p; hsa-miR-194-5p; hsa-miR-21-5p; hsa-miR-223-3p |
| 39 | Blood Clotting Cascade | 0.0223341 | hsa-miR-155-5p; hsa-miR-21-5p |
| 40 | Pantothenate and CoA biosynthesis | 0.0223341 | hsa-miR-155-5p; hsa-miR-21-5p |
| 41 | TGF beta Signaling Pathway2 | 0.0237601 | hsa-miR-155-5p; hsa-miR-194-5p; hsa-miR-21-5p; hsa-miR-223-3p |
| 42 | Oxidative Stress | 0.0238916 | hsa-miR-155-5p; hsa-miR-21-5p; hsa-miR-223-3p |
| 43 | Cysteine and methionine metabolism | 0.0247927 | hsa-miR-155-5p; hsa-miR-194-5p; hsa-miR-21-5p |
| 44 | MicroRNAs in cardiomyocyte hypertrophy | 0.0250118 | hsa-miR-155-5p; hsa-miR-194-5p; hsa-miR-21-5p; hsa-miR-223-3p |
| 45 | Glycosphingolipid biosynthesis lacto and neolacto series | 0.0257693 | hsa-miR-155-5p; hsa-miR-21-5p |
| 46 | Autoimmune thyroid disease | 0.0257693 | hsa-miR-155-5p; hsa-miR-21-5p |
| 47 | Endochondral Ossification | 0.0263091 | hsa-miR-155-5p; hsa-miR-194-5p; hsa-miR-21-5p; hsa-miR-223-3p |
| 48 | TNF alpha Signaling Pathway | 0.027653 | hsa-miR-155-5p; hsa-miR-194-5p; hsa-miR-21-5p; hsa-miR-223-3p |
| 49 | Plasminogen activating cascade | 0.0294184 | hsa-miR-155-5p; hsa-miR-21-5p |
| 50 | EBV LMP1 signaling | 0.0296024 | hsa-miR-155-5p; hsa-miR-21-5p; hsa-miR-223-3p |
| 51 | Transcription regulation by bZIP transcription factor | 0.0306259 | hsa-miR-155-5p; hsa-miR-194-5p; hsa-miR-21-5p |
| 52 | Endogenous cannabinoid signaling | 0.0313213 | hsa-miR-155-5p; hsa-miR-21-5p |
| 53 | EPO Receptor Signaling | 0.0327352 | hsa-miR-155-5p; hsa-miR-21-5p; hsa-miR-223-3p |
| 54 | Nicotinate and nicotinamide metabolism | 0.0332754 | hsa-miR-155-5p; hsa-miR-21-5p |
| 55 | NLR proteins | 0.03528 | hsa-miR-155-5p; hsa-miR-223-3p |
| 56 | Retinol metabolism | 0.03528 | hsa-miR-155-5p; hsa-miR-21-5p |
| 57 | Cytokine cytokine receptor interaction | 0.0359136 | hsa-miR-155-5p; hsa-miR-194-5p; hsa-miR-21-5p; hsa-miR-223-3p |
| 58 | PDGF signaling pathway | 0.0367405 | hsa-miR-155-5p; hsa-miR-194-5p; hsa-miR-21-5p; hsa-miR-223-3p |
| 59 | Integrated Breast Cancer Pathway | 0.0367405 | hsa-miR-155-5p; hsa-miR-194-5p; hsa-miR-21-5p; hsa-miR-223-3p |
| 60 | Progesterone mediated oocyte maturation | 0.0367405 | hsa-miR-155-5p; hsa-miR-194-5p; hsa-miR-21-5p; hsa-miR-223-3p |
| 61 | Alpha adrenergic receptor signaling pathway | 0.0373345 | hsa-miR-155-5p; hsa-miR-21-5p |
| 62 | Oocyte meiosis | 0.0375805 | hsa-miR-155-5p; hsa-miR-194-5p; hsa-miR-21-5p; hsa-miR-223-3p |
| 63 | RIG I like receptor signaling pathway | 0.0383759 | hsa-miR-155-5p; hsa-miR-21-5p; hsa-miR-223-3p |
| 64 | Signaling Pathway | 0.0395676 | hsa-miR-155-5p; hsa-miR-194-5p; hsa-miR-21-5p |
| 65 | Jak STAT signaling pathway | 0.0401802 | hsa-miR-155-5p; hsa-miR-194-5p; hsa-miR-21-5p; hsa-miR-223-3p |
| 66 | Cell adhesion molecules CAMs | 0.0407806 | hsa-miR-155-5p; hsa-miR-194-5p; hsa-miR-21-5p |
| 67 | Aldosterone regulated sodium reabsorption | 0.0407806 | hsa-miR-155-5p; hsa-miR-21-5p; hsa-miR-223-3p |
| 68 | Metabotropic glutamate receptor group I pathway | 0.0415897 | hsa-miR-155-5p; hsa-miR-21-5p |
| 69 | Graft versus host disease | 0.0415897 | hsa-miR-155-5p; hsa-miR-21-5p |
| 70 | B Cell Receptor Signaling Pathway | 0.0429017 | hsa-miR-155-5p; hsa-miR-194-5p; hsa-miR-21-5p; hsa-miR-223-3p |
| 71 | Irinotecan Pathway | 0.0437891 | hsa-miR-155-5p; hsa-miR-21-5p |
| 72 | Valine leucine and isoleucine biosynthesis | 0.0437891 | hsa-miR-155-5p; hsa-miR-21-5p |
| 73 | Intestinal immune network for IgA production | 0.0437891 | hsa-miR-155-5p; hsa-miR-21-5p |
| 74 | FAS signaling pathway | 0.0445485 | hsa-miR-155-5p; hsa-miR-21-5p; hsa-miR-223-3p |
| 75 | FSH signaling pathway | 0.0458475 | hsa-miR-155-5p; hsa-miR-21-5p; hsa-miR-223-3p |
| 76 | Ether lipid metabolism | 0.0460354 | hsa-miR-155-5p; hsa-miR-21-5p |
| 77 | Notch signaling pathway | 0.0485105 | hsa-miR-155-5p; hsa-miR-194-5p; hsa-miR-21-5p |
| 78 | Insulin signaling pathway | 0.0487205 | hsa-miR-155-5p; hsa-miR-194-5p; hsa-miR-21-5p; hsa-miR-223-3p |
