# Supplementary figures and images for: Exploring the pathogenesis linking traumatic brain injury and epilepsy via bioinformatic analyses
Source: Front Aging Neurosci. 2022 Nov 10;14:1047908. doi: 10.3389/fnagi.2022.1047908 (PMC9686289; doi:10.3389/fnagi.2022.1047908)

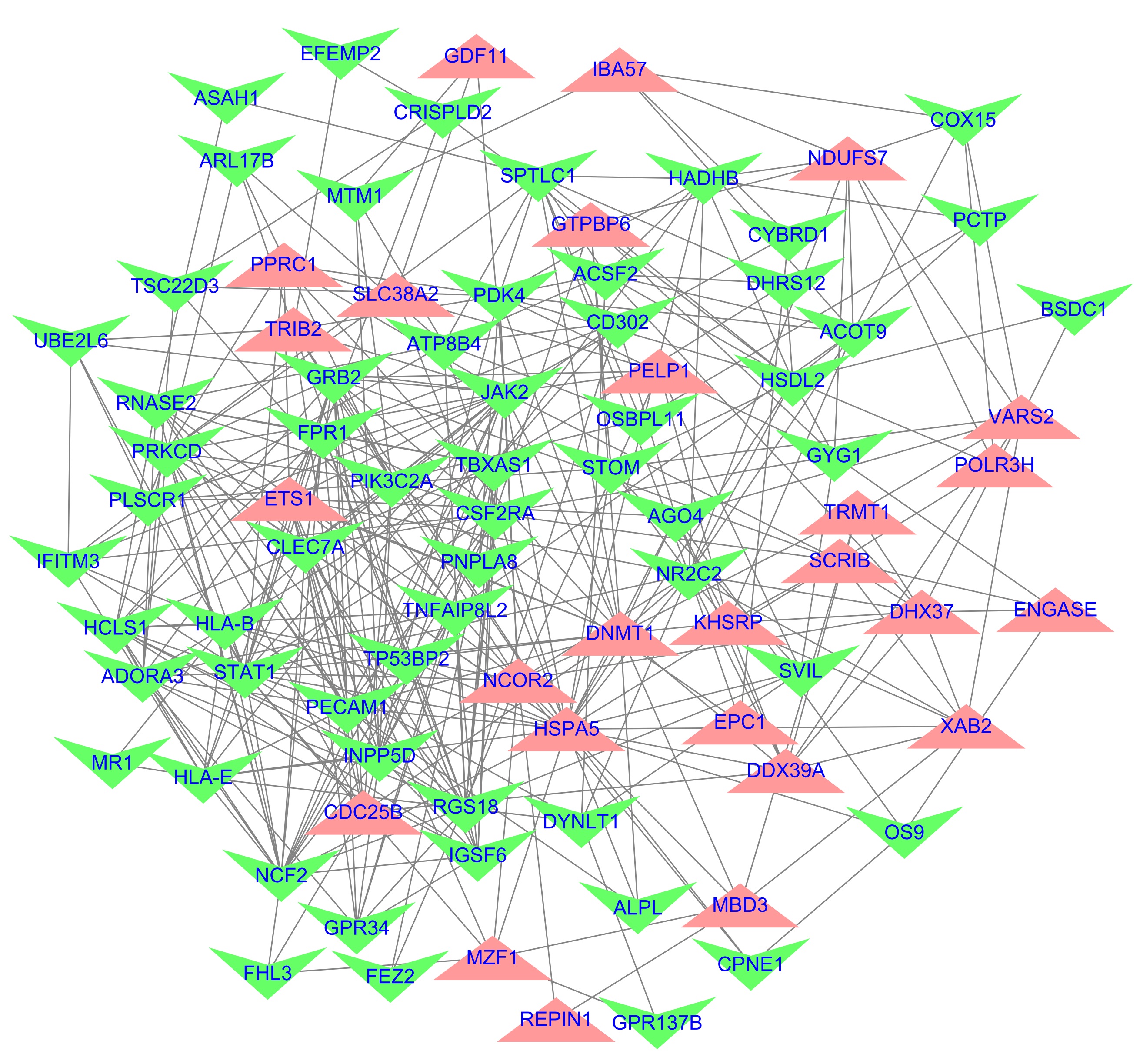

Supplement: Supplementary Figure 1 — Protein–protein interactions network of the cross-talk genes red indicates upregulated genes, and green indicates downregulated genes. [file Image_1.JPEG]

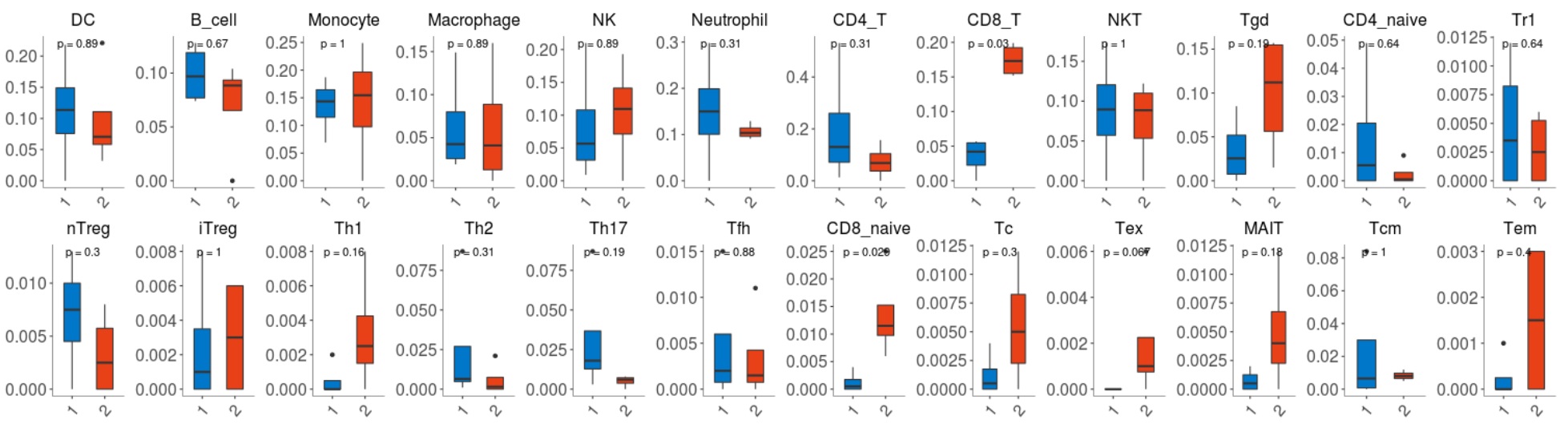

Supplement: Supplementary Figure 2 — Immune cell comparisons between PTE group and control group. P-value < 0.05 is considered statistically significant. The blue box plot indicates PTE group and the red box plot represents contRMAT 4. [file Image_2.JPEG]
